# Identification of Pathogenic *Leptospira kirschneri* Serogroup Grippotyphosa in Water Voles (*Arvicola terrestris*) from Ruminant Pastures in Puy-de-Dôme, Central France

**DOI:** 10.3390/pathogens12020260

**Published:** 2023-02-06

**Authors:** Elena Harran, Adrien Pinot, Angeli Kodjo, Zouheira Djelouadji, Marine Le Gudayer, Soro Sionfoungo Daouda, Karine Groud, Virginie Lattard, Florence Ayral

**Affiliations:** 1 USC 1233-RS2GP, VetAgro Sup, Université de Lyon, 69280 Marcy L’Etoile, France; 2 Faculty of Arts and Sciences, Holy Spirit University of Kaslik (USEK), Jounieh P.O. Box 446, Lebanon; 3 Laboratoire des Leptospires et d’Analyses Vétérinaires, VetAgro Sup, Université de Lyon, 69280 Marcy L’Etoile, France

**Keywords:** leptospirosis, water voles, maintenance hosts, reservoir, diagnosis, epidemiology

## Abstract

Rodents are the primary reservoirs for pathogenic *Leptospira* species, which cause leptospirosis. Among the key potential carriers are water voles, whose population outbreaks can consequently pose a major threat to human and animal health. We studied the prevalence, prominence, and epidemiology of pathogenic *Leptospira* species in water voles in central France. First, 46 voles were captured, and DNA was extracted from kidney, lung, liver, blood, and urine and tested for the presence of *Leptospira* using three molecular methods: PCR, O-antigen typing, and variable number tandem repeat (VNTR) typing. We also attempted to culture leptospires from kidney and urine samples. In addition, we investigated leptospiral antibodies in serum samples from 60 sheep using microscopic agglutination testing. These animals co-occurred with the voles, so we sought to assess their degree of exposure and involvement in pathogen dynamics. The overall prevalence of infection was 76.1% (CI_95%_ [61.2%, 87.4%]). The only strain found was *L. kirschneri* serogroup Grippotyphosa and a similar VNTR profile was acquired. Leptospires were successfully cultured from kidney and urine samples for four voles. Three sheep had low antibody titers against the *Leptospira* serogroup Grippotyphosa. Taken together, our results suggest the exclusive carriage of *L. kirschneri* serogroup Grippotyphosa among water voles in central France. Nevertheless, their ability to act as reservoir hosts that transmit the pathogen to co-occurring livestock remains unclear and merits further research.

## 1. Introduction

Leptospirosis is considered to be the world’s most widespread bacterial zoonosis and is responsible for 1.03 million cases of illness and 60,000 deaths annually [1,2]. The clinical manifestations of the disease depend on the host. For example, infections may be asymptomatic and highly persistent in rodents, lead to subclinical and chronic disease in domestic ruminants, or cause severe, potentially fatal illness in humans [2,3,4,5,6]. Transmission in humans and animals is mainly indirect, and incidence is higher in warm and humid climatic zones [7,8]. Global climate change is likely behind the recent increase in leptospirosis incidence and outbreaks [9,10], a trend that has been observed in the human population of mainland France since 2014 [11]. Awareness is thus growing that leptospirosis represents a significant threat to public health [12,13,14]. That said, little is known about many fundamental aspects of leptospirosis epidemiology, such as pathogen ecology and pathogen dynamics in wildlife hosts. Consequently, we have a limited understanding of disease drivers and effective prevention strategies.

The genus *Leptospira* currently comprises 68 species of highly diverse bacteria that form two clades—the pathogens and the saprophytes—that each, in turn, contain two subclades (P1 and P2 vs. S1 and S2); these groups differ in their virulence and genetic characteristics [15]. Among the pathogenic species, *L. interrogans, L. kirschneri,* and *L. borgpetersenii* predominate and circulate worldwide; *L. interrogans* is most commonly seen in individual cases of leptospirosis, while *L. kirschneri* is regularly associated with outbreaks in humans [5,16,17]. *Leptospira* species are categorized into serogroups that are further divided into serovars [18]. In epidemiological studies conducted in nature, it is essential to reliably characterize *Leptospira* serogroups via standardized laboratory tests [1] because there are strong associations between certain serogroups and hosts [5,19]. 

Pathogenic *Leptospira* species can infect all mammal species, but their dynamics and dissemination are host specific. For example, some mammals may act as maintenance hosts for particular serogroups, while others act as accidental hosts. In the latter group, the outcome of infection is either recovery or death. The pathogen does not naturally persist in accidental hosts; however, except in the case of humans, accidental hosts can contribute to ecosystem-level pathogen persistence if they co-occur with hosts of either type [20]. Conversely, maintenance hosts tend to be infected by particular serogroups that colonize the kidneys and are shed in the urine over long periods of time [21]. They may act as chronic selective carriers of particular *Leptospira* serogroups in a range of ecosystems and possibly transmit the pathogen to accidental hosts [1]. For example: brown rats (*Rattus norvegicus)* are thought to be selective carriers of the serogroup Icterohaemorrhagiae and cause accidental infections in humans [22,23]. Cattle (*Bos taurus*) are selective carriers of the serogroup Sejroe [24]; dogs (*Canis familiaris)* of the serogroup Canicola [25]; and swine (*Sus scrofa*) of the serogroups Australis and Pomona [5,26,27,28]. Although rats, cattle, dogs, and pigs might serve as maintenance hosts for other *Leptospira* serogroups, experimental research has yet to explore this question. Particular *Leptospira* serogroups persist within ecosystems thanks to specific host–pathogen relationships and ecological interactions, which means that ecosystems each possess their own set of maintenance mechanisms and levels of environmental contamination [20,29]. The hosts making the greatest contributions to these dynamics form the maintenance community and can end up infecting target species of concern [20,21]. Over the past 50 years, mainland France has witnessed dramatic shifts in land cover as a result of agricultural development, leading to decreased biodiversity [30]. In the current pasture ecosystems of central France, water voles (*Arvicola terrestris*) and domestic ruminants are the dominant fauna [31]. They co-occur and may share a number of epidemiological connections.

Outbreaks of water vole populations occur every 6 years on average, with densities peaking at 500–1000 individuals/hectare for 1–3 years [32,33]. If a vole population is headed toward an outbreak and is hosting a pathogenic *Leptospira* species, humans, livestock, and other animals in the same environment may be at risk [34]. Recently in Europe, the presence of *Leptospira* species has been reported in a variety of voles, such as common voles (*Microtus arvalis*), field voles (*Microtus agretis*), water voles (*Arvicola terrestris*), and bank voles (*Myodes glareolus*). These studies were mainly conducted in Germany and Spain and specifically identified *L. kirchneri, L. borgpeterseni*, and *L. interrogans* [35,36,37,38]. A handful of more detailed studies have been carried out on European water vole populations, including two in eastern France. One showed that water voles were carrying *Leptospira* species but did not identify any genetic profile [39]. The second found *L. kirschneri* to be the only species present [40]. However, research has yet to address the prevalence of *Leptospira* species in water voles in central France while also examining the voles’ potential role in transmitting leptospires to hosts such as domestic ruminants. This issue is particularly important to address given the regular occurrence of vole outbreaks. 

Our study’s major aim was to investigate the potential role of water voles and sheep in *Leptospira* epidemiology in pasture ecosystems in central France. To this end, our specific objectives were as follows: (1) characterize *Leptospira* prevalence in vole populations during outbreaks; (2) determine whether leptospires were present in vole tissues to clarify potential infection type (chronic vs. acute) and transmission pathways; (3) obtain complete genetic profiles, namely serogroup identity, and assess whether selective carriage could be occurring; and (4) ascertain whether water voles might transmit leptospires to co-occurring sheep.

## 2. Materials and Methods

### 2.1. Ethics Statement on Vole Sampling

The authors confirm that all the research described herein complied with national and institutional regulations related to the care and use of animals (APAFiS, no. 37713, project authorization no. 2238). 

### 2.2. Provision of Sheep Blood Samples

Sheep blood samples collected for annual herd prophylaxis program were used in this study after obtaining owner consent.

### 2.3. Sampling Sites

**Figure 1:**
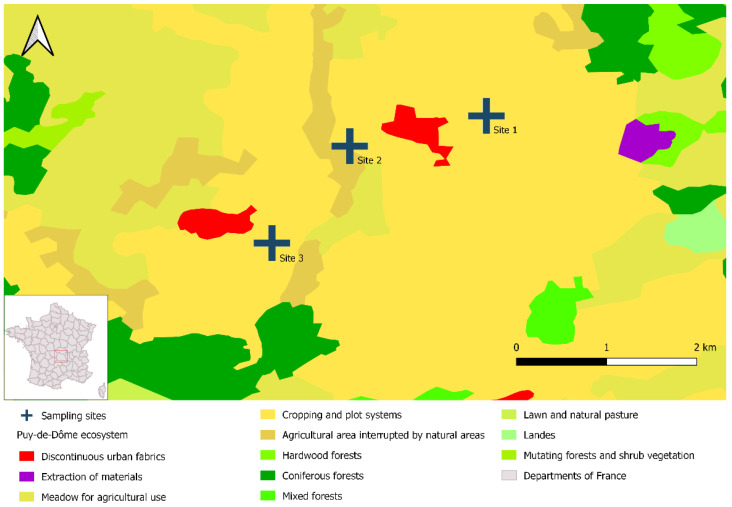
Location of the three study sites in Puy-de-Dôme, France. The map was created with DIVA-GIS (v. 7.5) and data from Corine Land Cover (2018 Edition, mainland France); it was designed with QGIS 3.16.1 Hannover.

We trapped water voles in three different livestock breeding sites over a single day in November 2021. The pastures were separated by 1.4 to 1.6 km and were located in the French administrative department of Puy-de-Dôme. This department is in central France and nearly 50% of its surface area (400,000 ha) is dedicated to agriculture, mainly in the form of grasslands [41]. Vole outbreaks are common in Puy-de-Dôme; population densities can exceed 500 voles/ha [42,43]. We selected these three study sites because they had large vole populations and displayed specific environmental characteristics, notably agricultural lands with crops organized into plot systems (Figure 1).

### 2.4. Vole Trapping and Sampling

Voles were live captured using tube traps and lethally captured using Topcat traps. The 26 living voles were immediately anaesthetized using isoflurane and euthanized via cardiac puncture and cervical dislocation. They were quickly dissected to take tissue samples, which were then stored at −20 °C for 4 weeks, at which point the molecular analyses were conducted. These animals are hereafter referred to as the euthanized voles. Twenty voles were lethally captured. Their entire bodies were immediately frozen and then stored at −20 °C. Dissection and tissue sampling occurred 10 weeks later and were immediately preceded by a 24-h thawing period. These animals are hereafter referred to as the cold-stored voles. For each vole, we noted the following: sex (presence of genital tract = females, presence of testes/penis = male), length (body = from nose to anus and overall = from nose to tail), and state of sexual maturity (developed uterus for females, presence of seminal vesicles for males). 

For both groups, dissection and tissue sampling took place as follows. Three sets of dissecting instruments were alternated during these processes. To prevent cross-contamination, the instruments were regularly cleaned and disinfected, notably between tissue sampling within animals and between dissections across animals. When possible, samples of kidney, lung, liver, blood, and urine were collected for each animal. Kidney and lung samples were obtained to evaluate evidence of renal colonization and pulmonary carriage, respectively; the latter has been described in rats [44]. Blood and liver samples were obtained to assess the occurrence of acute septicemic infection, and urine was obtained to evaluate the possibility of leptospire excretion. All the samples were stored individually in 2-mL Eppendorf tubes at −20 °C until the molecular analyses could be carried out.

### 2.5. Sheep Sampling 

Serological diagnosis is considered adequate to define *Leptospira*-status among sheep herds and determine circulating leptospiral antibodies (with antibodies persisting several months) by the World Organization for Animal Health (WOAH) [45]. At least 10% of the herd was sampled, in order to gather relevant data at the herd level as recommended by the WOAH [45]. 

Site 3 hosted a herd of nearly 200 sheep. Consequently, samples were taken from 2 batches of 30 sheep at 2 time points: 6 months after the grazing period and 6 months after the initial sampling period, which was 2 weeks immediately after the next grazing period. 

### 2.6. Culturing Leptospires from Urine and Renal Tissue

We attempted to culture *Leptospira* bacteria from urine and kidney samples using Ellinghausen–McCullough–Johnson–Harris (EMJH) medium. First, EJMH and EJMH STAFF media were prepared under sterile conditions, as described elsewhere [46]. Briefly, a volume of 1 mL of urine was collected and a piece of fresh kidney tissue of about 1 cm^3^ was crushed inside the tube of a sterile syringe of 5 mL using the plunger. A volume of 1 mL of both preparations was independently added to a first tube containing EJMH STAFF medium. The tubes were vortexed for a few seconds, yielding a 1/10 dilution. Next, 1 mL of the diluted solutions was transferred to a second set of tubes that contained EJMH medium. These tubes were vortexed for a few seconds, yielding a 1/100 dilution. The tubes were then incubated at 30 °C. Over a two-month period, we regularly assessed the presence or potential growth of leptospires on the medium via dark-field microscopy (DFM) [47]. If no leptospires were observed during that period, culture was considered negative.

### 2.7. Extraction and Detection of Leptospira DNA

DNA was isolated from kidney, lung, liver, blood and urine using a DNeasy Blood and Tissue Kit (Qiagen, Hilden, Germany) and from successful cultures (containing at least 5 × 10^6^ cells) using a QIAamp DNA Mini Kit (Qiagen, Courtaboeuf, France). In both cases, we followed the manufacturer’s instructions. The quantitation of the β-actin endogenous housekeeping gene using real time PCR (RT-PCR) was used to assess the efficiency of DNA extraction and the absence of inhibitors for each sample and served as an internal control for the target gene expression [48]. RT-PCR targeting the 16S rRNA gene was performed using AgPath-ID™ One-Step RT-PCR Reagents (Applied Biosystems) and specific primers previously described to detect for pathogenic *Leptospira* species [49]. The PCR conditions were as follows: 10 min at 95 °C as the denaturation step, and 40 cycles of (a) 15 s at 95 °C as the amplification step and (b) 1 min at 60 °C as the annealing step. Each run included a negative control (the PCR mix without the target DNA) and a positive control (DNA from the *L. interrogans* serovar Icterohaemorrhagiae ENVN strain). When a sample had a cycle threshold (C_T_) that was equal to or less than 40, it was considered to be positive for *Leptospira*.

### 2.8. Genetic Characterization of Leptospira

#### 2.8.1. Conventional PCR Targeting the 16S rDNA Gene

Samples found to be positive for *Leptospira* via RT-PCR were then subject to conventional PCR (cPCR) targeting the 16S rDNA gene, using specific primers described elsewhere [50]. We used a HotStarTaq DNA Polymerase Kit (Qiagen, Courtaboeuf, France) and the following cPCR conditions: 15 min at 95 °C as the first denaturation step; 40 cycles of (a) 15 s at 95 °C for the second denaturation step, (b) 30 s at 57 °C as the annealing step, and (c) 1 min at 72 °C as the initial elongation step; and 10 min at 72 °C as the final elongation step. Each run included a negative control (the PCR mix without the target DNA) and a positive control (DNA from the *L. interrogans* serovar Icterohaemorrhagiae ENVN strain). The amplified products were verified utilizing electrophoresis on 1% agarose gel (30 min at 100 v). Under ultraviolet light (UV) conditions, the products’ molecular weights were assessed via comparisons with a 100-bp ladder (Invitrogen) and the positive control. The products were then subject to Sanger sequencing (performed by Genoscreen, Lille, France). ChromasPro (v. 2.6.6) was used to assemble the sequences, creating contigs. We then identified the *Leptospira* species present using an NCBI Nucleotide BLAST search (http://blast.ncbi.nlm.nih.gov, (accessed on 1 November 2022)). 

#### 2.8.2. Molecular Typing of Leptospira DNA Based on O-Antigen and Variable Number Tandem Repeat (VNTR) Methods

To identify the *Leptospira* strains present, we used two forms of molecular typing, one using O-antigen and one using VNTR. O-antigen typing was performed on positive samples (C_T_ ≤ 40) obtained from renal tissue and successful cultures, using primers conceived in previous article [51]. The cPCR conditions were as follows: 15 min at 95 °C as the first denaturation step; 30 cycles of (a) 30 s at 94 °C as the second denaturation step, (b) 30 s at 60 °C, and (c) 1 min at 72 °C as the initial extension step; and 10 min at 72 °C as the final extension step. Each run included a negative and a positive control. The PCR mix without the target DNA was the negative control. DNA from the *L. kirschneri* serovar Grippotyphosa Moskva V strain and from the *L. interrogans* serovar Icterohaemorrhagiae ENVN strain were the positive controls; they allowed serogroup differentiation. The amplified products were verified utilizing electrophoresis on 1% agarose gel (30 min at 100 v). Under UV conditions, the products’ molecular weights were assessed via comparisons with a 100-bp ladder (Invitrogen) and the positive control.

VNTR typing was performed on positive samples (C_T_ < 35) obtained from renal tissue and successful cultures. VNTR-4, VNTR-7, and VNTR-10 loci were amplified using primers described elsewhere [52]. The cPCR conditions were as follows: 15 min at 95 °C as the first denaturation step; 40 cycles of (a) 30 s at 95 °C as the second denaturation step, (b) 30 s at 54 °C (VNTR-4/VNTR-7) or 52 °C (VNTR-10) as the annealing step, (c) 1 min at 72 °C as the initial extension step; and 10 min at 72 °C as the final extension step. Each run included a negative control as well as VNTR-specific positive controls: there were 4 controls for VNTR-4 (copies 0-1-2-3), 2 controls for VNTR-7 (copies 1 and 2), and 4 controls for VNTR-10 (copies 4-7-11-12). The amplified products were verified utilizing electrophoresis on 1% agarose gel (30 min at 100 v). Under UV conditions, the products’ molecular weights were assessed via comparisons with a 100-bp ladder (Invitrogen) and the positive controls. Band sizes were used to deduce the copy number of the repeats relative to each VNTR and, thus, establish a VNTR profile for each sample.

### 2.9. Microagglutination Testing

Microscopic agglutination test (MATs) were carried out on the sheep’s blood samples using a panel of live isolated leptospires. We considered a series of 12 serogroups (and their associated serovars): Australis (Australis, Bratislava, Munchen), Autumnalis (Autumnalis, Bim), Ballum (Castellonis), Bataviae (Bataviae), Canicola (Canicola), Grippotyphosa (Grippotyphosa, Vanderhoedoni), Icterohaemorrhagiae (Icterohaemorrhagiae, Copenhageni), Panama (Panama, Mangus), Pomona (Pomona, Mozdok), Pyrogenes (Pyrogenes), Sejroe (Sejroe, Saxkoebing, Hardjo, Wolffi), and Tarassovi (Tarassovi) (Table S1). Leptospire agglutination was assessed using DFM. Any samples with an agglutination level of at least 50% were further diluted to establish the titer endpoint for each of the 22 serovars tested. A titer of 1:100 was used as the cut-off threshold for seropositivity, as per WOAH guidelines [45]. MAT results were analyzed at the serogroup level [53]. The putative serogroup responsible for an infection was identified when a titer was obtained against one or more serovars in a given serogroup or when the maximum titer against a given serogroup was at least threefold higher than those against any other serogroups. We classified samples as displaying equal dominance when they reacted to two or more serogroups but there was less than a threefold difference in titers. In such instances, the result was deemed to be inconclusive; such is a frequent outcome given cross reactions.

### 2.10. Data Analysis

A vole was considered to be infected with *Leptospira* if at least one specimen was tested positive. The characteristics of infected and uninfected voles were compared using Rstudio (v. 1.3.1093, Apricot Nasturtium). An ANOVA (t.test function) was performed to evaluate the influence of length on infection status. A Chi-square test (chisq.test function) was used to assess the influences of sex, sexual maturity, category, and study site on infection status, including the estimate of *p*-value to consider small populations. The 95% confidence intervals for prevalence were calculated using the binom.test function.

## 3. Results

### 3.1. Vole Characteristics

**Figure 2:**
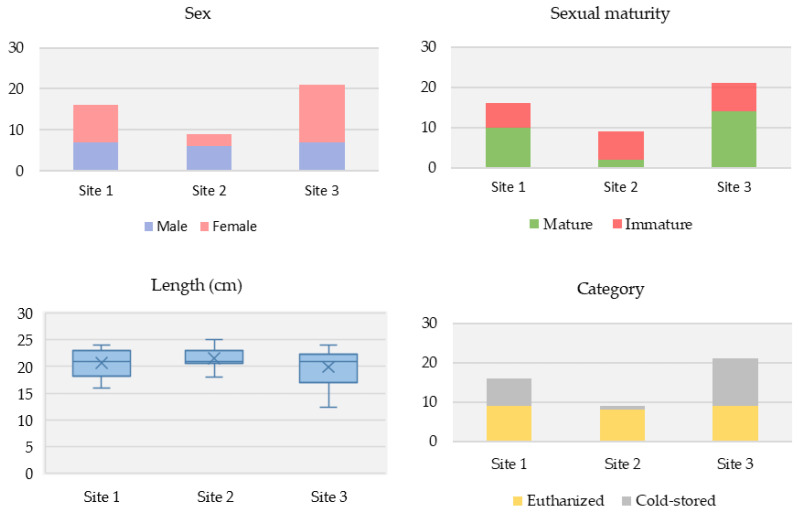
Differences in vole characteristics across the three Puy-de-Dôme study sites. (**Top left**): Sex. (**Top right**): Sexual maturity status. (**Bottom left**): Overall average length. (**Bottom right**): Sampling category.

Among the voles sampled, there were more females than males (56.5% [26/46] vs. 43.5% [20/46], respectively). Over half had reached sexual maturity (56.5% [26/46]). Their average body length (nose to anus) was 15 cm, and average overall length (nose to tail) was 20.5 cm. Vole characteristics differed among the three study sites (Figure 2).

Most voles displayed an absence of morphological abnormalities in their tissues. Just three euthanized voles were found to have liver cysts. Additional information is available in Table S2.

### 3.2. Vole Infection Status

#### 3.2.1. Cultures

Leptospires were successfully cultured from the tissues (*n* = 4 kidney samples, *n* = 1 urine sample) of four euthanized voles.

#### 3.2.2. Characteristics of Infected Voles

Based on the RT-PCR results for the kidney samples, infection status was unrelated to vole sex, sexual maturity status, or site of origin. However, euthanized voles were more likely than cold-stored voles to be infected; the same was true for voles with greater overall lengths (Table 1).

#### 3.2.3. Detecting Leptospira Infection in Voles

**Figure 3:**
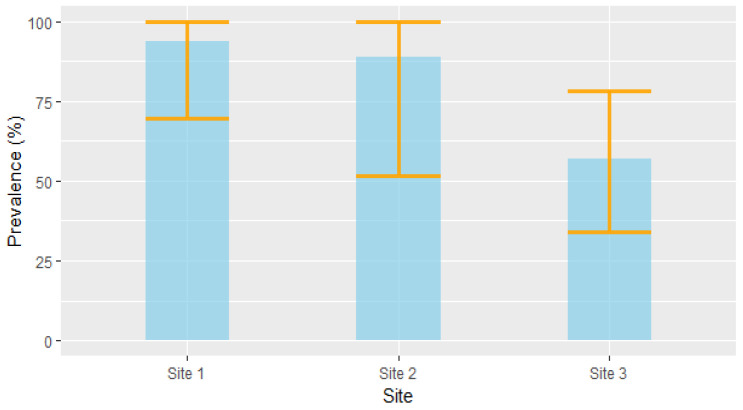
Prevalence (±CI_95%_) of Leptospira infection in voles across the three study sites.

Based on the RT-PCR results, 35 of the 46 voles were positive for *Leptospira* DNA, resulting in an overall prevalence of 76% (CI_95%_ [61%, 87%]). Although the differences were not statistically significant, prevalence tended to vary among the study site: site 1 had a prevalence of 93.7% (CI_95%_ [69.7%, 99.8%], *n* = 16); site 2 had a prevalence of 88.9% (CI_95%_ [51.7%, 99.7%], *n* = 9); and site 3 had a prevalence of 57.1% (CI_95%_ [34%, 78,2%], *n* = 21) (Figure 3).

**Figure 4:**
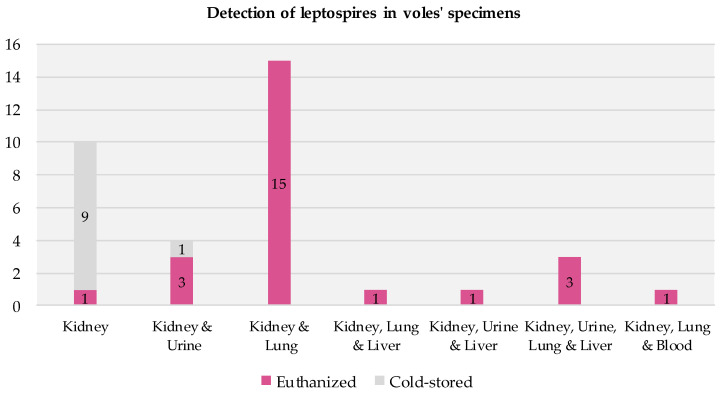
Presence of infection across specimens in voles testing positive for Leptospira via RT-PCR.

All the voles that tested positive had positive kidney samples. In most cases, their other specimens (urine, lung, liver, and blood) also tested positive (Figure 4). Among the 25 positive euthanized voles, all but one had multiple infected specimens. Among the 10 positive cold-stored voles, infections were detected solely in the kidney specimens (9/10) or in both the kidney and urine specimens (1/10).

#### 3.2.4. Molecular Typing of Leptospira Strains in Voles

We obtained the predicted 330-bp fragments when we conducted cPCR on *Leptospira* DNA obtained from the vole specimens (i.e., kidney, lung, liver, blood, and urine) and successful cultures that had tested positive via RT-PCR (C_T_ ≤ 40); the positive controls confirmed result reliability [54]. In the BLAST search, all the sequences displayed high nucleotide affinity (97–100%) with *L. kirschneri* (GenBank accession number MK726123.1).

O-antigen typing was applied to DNA obtained from kidney samples (*n* = 19 samples) and successful cultures (*n* = 4) that had tested positive via RT-PCR (C_T_ ≤ 40); the positive controls confirmed result reliability. Evidence was found for the presence of the *Leptospira* serogroup Grippotyphosa (GRIP) but not for the *Leptospira* serogroup Icterohaemorrhagiae (IH).

Two VNTR profiles were observed in the DNA obtained from the kidney samples. The 1-2-11 profile was complete and had 1 TR copy at the VNTR 4 locus, 2 TR copies at the VNTR 7 locus, and 11 TR copies at the VNTR 10 locus. The 1-2-X profile was incomplete and had 1 TR copy at the VNTR 4 locus, 2 TR copies at the VNTR 7 locus, and no observable amplification at the VNTR 10 locus. The first profile was seen in 9 of the 14 samples, and the second profile was seen in 5 of the 14 samples. The first VNTR profile was also seen in all four of the DNA samples from the successful cultures. A summary of the molecular typing results for the DNA obtained from the kidney samples is provided in Table 2.

### 3.3. Leptospira Seroprevalence in Sheep

#### Microagglutination Testing

In the first sampling round, four sheep were seropositive (MAT titers ≥ 1:100). Their reactions to the *Leptospira* serogroups were as follows: two reacted to IH (titer values = 1:100); one reacted to IH and GRIP (both titer values = 1:100); and one reacted to Sejroe (SJ) (titer value = 1:200). 

In the second sampling round, two sheep were seropositive (MAT titers = 1:100), and both reacted to GRIP.

## 4. Discussion

This study shows the important and potential exclusive carriage of *L. kirschneri* serogroup Grippotyphosa in water vole populations across three Puy-de-Dôme pastures, as well as the seroconversion of sympatric sheep likely exposed to *Leptospira* serogroup Grippotyphosa following their grazing on site 3. 

*Leptospira* infections in water voles

Across all three water vole populations, the prevalence of *Leptospira* infection was 76% (CI_95%_ [61%, 87%]). In infected animals, pathogenic *Leptospira* DNA was consistently found in the kidneys, which is unsurprising since these organs are frequently colonized by leptospires [1,55,56]. What is surprising is that this prevalence is markedly higher than that seen in *R. norvegicus* in France (26%, CI_95%_ [20%, 33%]) [57], an intriguing result because *R. norvegicus* is considered to be the primary reservoir host for the pathogen causing human leptospirosis [23]. Such findings underscore that *Leptospira* prevalence can be extremely high in water vole populations, which highlights the need to explore their role in the epidemiology of leptospirosis. 

If anything, it seems likely that 76% was an underestimate given that 20 of the 46 voles making up our sample belonged to the cold-stored group, which was less likely than the euthanized group to test positive for infection. This pattern may stem from methodological differences. The euthanized voles immediately underwent dissection and tissue sampling, and the molecular analyses occurred after just four weeks of sample cold storage. In contrast, the cold-stored voles went about ten weeks before dissection and tissue sampling were conducted. DNA concentrations start to decline after such lengths of time [58] because freezing affects bacterial richness and abundance; consequently, the likelihood of detecting any *Leptospira* DNA probably fell [59,60,61]. In addition, compared to the euthanized voles, the cold-stored voles experienced one additional cycle of freezing/thawing prior to the molecular analyses. It is known that the number of freezing/thawing cycles can cause DNA degradation and reduce amplification success, leading to a higher probability of false negatives [62,63,64]. Finally, the thawing period experienced by the cold-stored voles could have allowed the intestinal microbiota to trickle through the permeable intestinal tissue. If leptospires were present, their concentrations could have been diluted by this other source of bacteria, reducing the detectability of *Leptospira* DNA. That said, no such phenomenon has ever been reported in the literature.

In past research, certain rodent characteristics have been associated with infection status. Indeed, females are at greater risk of *Leptospira* infections than are males [65,66,67]. Furthermore, sexually mature rodents have a higher probability than juveniles of acquiring the pathogen because they can experience direct transmission during copulation and they spend more time exploring the outer environment [68]. Our study’s small sample size (*n* = 46) might be inadequate for testing whether sex and sexual maturity influence infection status in water voles.

Evidence of chronic *Leptospira* infection in water voles

To better understand how water voles contribute to *Leptospira* epidemiology in pasture ecosystems, it is necessary to determine whether they experience acute or chronic infection. Indeed, if the infection is chronic and has a limited impact on host health, the bacteria could more easily be maintained in the kidneys and shed over prolonged periods of time, such as occurs in rats [22]. The tissue infection patterns we observed provide insight into this question. None of the voles we captured displayed morphological abnormalities (e.g., nephritis or hemorrhaging) that could be attributed to *Leptospira* [69,70]. At the same time, leptospires were clearly present in one or more of the voles’ tissues. Out of the 35 infected voles, 14 had positive kidney or kidney and urine samples but negative lung, liver, and blood samples. Such results suggest that bacterial presence is long lasting and stable in the kidneys. This pattern is seen in chronic infections in rats: leptospires remain in the kidneys long after they have been cleared from other organs (~one week post infection) [71]. In the other infected voles, *Leptospira* was simultaneously present in multiple tissues. The latter was unlikely to be evidence of an acute infection because leptospires were absent from the blood in all but one case.

Potential modes of *Leptospira* transmission in water voles

To assess whether voles could shed *Leptospira* in their urine, it is necessary to evaluate whether leptospires in the urine are viable, which can be assessed by culturing bacteria from urine samples [72,73]. In the case of one infected animal (euthanized vole 10), we obtained a successful culture from both urine and kidney samples. This finding provides evidence of leptospire viability and suggests that the other voles with positive urine samples could have been shedding viable *Leptospira*. Overall, samples from 4 of the 26 euthanized voles gave rise to successful cultures, even though *Leptospira* bacteria are notoriously challenging to isolate because of their slow growth [74]. One reason that successful cultures were not obtained for the other infected voles could relate to pathogen concentrations. The greater the pathogen concentration in a sample, the lower the C_T_ value and the higher the probability of obtaining a successful culture [75,76]. Indeed, kidney and urine samples with C_T_ values exceeding 22 never resulted in successful cultures. These results indicate that culturing is a far less sensitive technique than is PCR, as highlighted by numerous studies [77,78].

Observations supporting selective *Leptospira* carriage by water voles

In our study, genotype related to serogroup GRIP was reported for most of the infected voles (expect for those for which C_T_ ≥ 34) through O-antigen typing, a finding that suggests selective carriage is occurring.

The VNTR results provide additional support for this idea. Nine of the 14 voles with low C_T_ values (≤28) were found to carry *Leptospira* with the same genetic profile: 1 TR copy at VNTR4; 2 TR copies at VNTR7; and 11 TR copies at VNTR10. Five voles with higher C_T_ values (range: 29.8–34.4) carried *Leptospira* with a similar but incomplete profile: 1 TR copy at VNTR4; 2 TR copies at VNTR7; and X TR copies at VNTR10. Voles are territorial and live in colonies whose members display restricted movements. Therefore, a given population is likely to maintain a given *Leptospira* strain. We hypothesize that the incomplete second profile is actually the same as the first. Our second profile is the same as an incomplete *L. kirschneri* profile obtained from voles in eastern France [40]; as such, it might also be identical to our complete genetic profile. These findings provide support for the notion that even voles in distant ecosystems selectively carry *Leptospira* with similar genetic profiles. The complete profile we obtained does not match up with any of the profiles described previously [52], which may suggest it is an unreported profile for the serogroup GRIP. Future research should use next-generation sequencing to completely characterize the genomes of these strains. The results should reveal the regions that best differentiate the strains, allowing for easier identification.

Role of water voles in *Leptospira* epidemiology

O-antigen and VNTR typing both yielded support for the hypothesis that voles selectively carry and maintain *Leptospira* related to the serogroup GRIP. Thus, the pathogen is likely present in large concentrations in pasture soils. The MATs revealed that 3 of the 60 sheep tested had antibodies (titer = 1:100) against the serogroup GRIP, suggesting they had previously been infected by the latter serogroup. Previous research using MATs found similarly low antibody titers in sheep [79,80,81]. We might have expected higher seropositivity in the sheep given the markedly high pathogen prevalence in the water voles. Furthermore, Puy-de-Dôme experiences climatic conditions (e.g., regular rainfall and stable relative humidity) that should promote the survival of any leptospires present in diluted animal urine [82,83]. However, it may be that sheep rarely come in contact with water vole urine. In grasslands, most voles remain underground in burrows that are permanently sealed, although they may occasionally open up during dry periods; as a result, the animals rarely come up to the soil surface [84]. It is also possible that sheep display a low rate of seroconversion following infection [5]. Consequently, the frequency of transmission between water voles and sheep remains unclear, as does the degree to which sheep may be accidental hosts. Beyond understanding that water voles are maintenance hosts for *L. kirschneri* serogroup GRIP, we know little about the role played by these rodents in leptospirosis epidemiology. 

Studying the water vole’s predators could be instructive. Research in mainland France characterized the *Leptospira* bacteria behind renal infections in various wildlife species and discovered that genetic profile diversity was greater in species that prey upon rodents [85]. Notably, *L. kirshneri* serogroup GRIP was detected in certain mustelids, while foxes (*Vulpes vulpes*) tended to harbor *L. interrogans*. In contrast, no wolves (*Canis lupus*) were found to be infected. Furthermore, other studies found that canines were seropositive for the serogroup GRIP in France [86], as were wolves in Italy [87]. It is evident that further research is needed to clarify how the water vole’s predators might contribute to *Leptospira* transmission.

Over recent years, several leptospirosis outbreaks in human populations in France have been linked to *L. kirschneri* associated to the serogroup GRIP [14,88]. In addition, these same *Leptospira* strain have been found to cause accidental infections in domestic animals [89,90,91]. To date, a single study has sought to characterize the genetic profiles of pathogenic *Leptospira* serogroups in wild mammals in France: none of the animal species tested appeared to be a maintenance host for the serogroup GRIP [85]. Consequently, none of them seem to be responsible for cases of leptospirosis provoked by this *Leptospira* serogroup. Given that our results strongly support the water vole’s role as a maintenance host of *L. kirschneri* serogroups GRIP, they can inform future work tracking the sources of *Leptospira* infections in humans and domestic animals.

## 5. Conclusions

This study found a high prevalence of *Leptospira* infection in water vole populations in central France. Across infections and sites, a single profile emerged: *L. kirschneri* serogroup GRIP. This finding suggests that water voles selectively carry this *Leptospira* strain, an idea supported by results from RT-PCR, cPCR, O-antigen typing, and VNTR typing. At the same time, the water vole’s contribution to infections in domestic animals remains undetermined given the low seroprevalence observed in sheep co-occurring in the same habitat. It is important to take this research further because the water vole is the first species to be identified as a selective carrier of *L. kirschneri* serogroup GRIP. To clarify the epidemiology of leptospirosis in France, additional studies should be conducted on voles as well as on the wild and domestic animals that share the same habitat.

## Figures and Tables

**Table 1:** Relationship between infection status (positive vs. negative) and vole characteristics based on the RT-PCR results for kidney samples.

Characteristics	Subcategory	Total (%) (*n* = 46)	Positive (%) (*n* = 35)	Negative (%) (*n* = 11)	*p*-Value *
Sex	Male	20 (43.5)	15 (42.9)	5 (45.4)	1
	Female	26 (56.5)	20 (57.1)	6 (54.5)	
Sexual maturity	Mature	26 (56.5)	22 (62.9)	4 (36.4)	0.2
	Immature	20 (43.5)	13 (37.1)	7 (63.6)	
Sampling category	Euthanized	26 (56.5)	25 (71.4)	1 (9.1)	0.001
	Cold-stored	20 (43.5)	10 (28.6)	10 (90.9)	
Site	Site 1	16 (34.8)	15 (42.9)	1 (9.1)	-
	Site 2	9 (19.6)	8 (22.9)	1 (9.1)	1
	Site 3	21 (45.6)	12 (34.2)	9 (81.8)	0.3
Overall length (cm)	Median (Q1–Q3)	21 (18.25–22.6)	21 (19.5–22.8)	17 (16.5–21.8)	0.03

* determined using the most appropriate test (Chi-squared test or Anova exact test).

**Table 2:** Molecular results for *Leptospira* DNA extracted from renal tissue.

Voles	RT-PCR Positive	Presence of *L. kirschneri*	O-Antigen—GRIP	VNTR Profile 1-2-11	VNTR Profile 1-2-X
Nb/Nbt	35/46	35/35	19/35	9/14 ^†^	5/14 ^†^

Nb, number of voles testing positive; Nbt, total number of voles tested; RT-PCR, real time PCR; GRIP, Grippotyphosa; VNTR, variable number of tandem repeat; ^†^, positivity threshold: C_T_ < 35.

## Data Availability

The data presented in this study are provided in the main text and the Supplementary Materials.

## Supplementary Materials

The following supporting information can be downloaded at https://www.mdpi.com/article/10.3390/pathogens12020260/s1—Table S1: List of the reference strains employed in the MAT antigen panel; Table S2: Vole characteristics across the study sites and the RT-PCR results; and Table S3: Antibody titers against different *Leptospira* serovars in sheep.
